# Retinal Degeneration in Alzheimer’s Disease 5xFAD Mice Fed DHA-Enriched Diets

**DOI:** 10.3390/cells15010008

**Published:** 2025-12-19

**Authors:** Mário S. Pinho, Husaifa Ahfaz, Sandra Carvalho, Jorge Correia, Maria Spínola, José M. Pestana, Narcisa M. Bandarra, Paula A. Lopes

**Affiliations:** 1 CIISA—Centro de Investigação Interdisciplinar em Sanidade Animal, Faculdade de Medicina Veterinária, Avenida da Universidade Técnica, 1300-477 Lisboa, Portugal; mpinho@fmv.ulisboa.pt (M.S.P.); huzaifaali.ahfaz@autonoma.cat (H.A.); sandracarvalho@fmv.ulisboa.pt (S.C.); jcorreia@fmv.ulisboa.pt (J.C.); mariaspinola@fmv.ulisboa.pt (M.S.); miguelpestana01@gmail.com (J.M.P.); 2 Laboratório Associado para Ciência Animal e Veterinária (AL4AnimalS), Avenida da Universidade Técnica, 1300-477 Lisboa, Portugal; 3 Divisão de Aquacultura, Valorização e Bioprospeção (DivAV), Instituto Português do Mar e da Atmosfera (IPMA, I.P.), Avenida Alfredo Magalhães Ramalho, 6, 1495-165 Algés, Portugal; narcisa@ipma.pt; 4 CIIMAR—Centro Interdisciplinar de Investigação Marinha e Ambiental, Universidade do Porto, Rua dos Bragas 289, 4050-123 Porto, Portugal

**Keywords:** Alzheimer’s disease, 5xFAD mice, DHA-enriched diets, retinal health, retinal layers thickness, immunohistochemistry

## Abstract

**Highlights:**

1. The adequate intake of docosahexaenoic acid (DHA, 22:6n − 3) is important for preventing cognitive decline and assuring brain optimal function, especially in early Alzheimer’s disease (AD) stages.
2. The pivotal role of DHA for protecting retinal integrity in AD remains elusive.
3. The neuroprotective effects of DHA supplementation in 5xFAD mice were found in the retina, from both fish oil and DHASCO commercial oil, concerning the retinal layer thickness, TAU protein aggregates, and retinal ganglion cell layer density, respectively.
4. These retinal alterations promoted by dietary DHA reinforce the retina as a valuable site for detecting and monitoring AD.

**Abstract:**

Alzheimer’s disease (AD) is marked by cognitive decline, and also by retinal degeneration. Having in mind that docosahexaenoic acid (DHA, 22:6n − 3) is a safe, low-cost, and pivotal fatty acid for brain health and sustained cognitive function, this study exploits environmentally friendly non-fish sources as potential dietary supplements enriched with DHA to prevent or reverse AD. Forty 5xFAD transgenic male mice, aged five weeks old, were randomly distributed by five body weight-matched dietary groups (with eight animals each) and fed isocaloric diets based on the AIN-93M standard formulation for rodents for 6 months. Except for the control feed (without supplementation), each diet contained a modified lipidic fraction supplemented with 2% of the following: (1) linseed oil (LSO, rich in alpha-linolenic acid (ALA, 18:3n − 3)); (2) cod liver oil (fish oil, FO, rich in both DHA and eicosapentaenoic acid (EPA, 20:5n − 3)); (3) *Schizochytrium* sp. microalga oil (Schizo, with 40% of DHA); and (4) commercial DHASCO (DHASCO, with 70% of DHA). The aim of this study was to measure retinal neural layer thickness, calculate ganglion cell layer (GCL) density, and assess retinal injury by means of immunohistochemical staining for β-amyloid plaques deposition, TAU protein levels, and IBA1, as hallmark features of AD progression, in order to elucidate the effects of different dietary DHA treatments in Alzheimer’s retinas. Although no statistical differences were observed across retinal layer thicknesses depending on the diet (*p* > 0.05), there was a consistent pattern for slightly increased retinal thickness in 5xFAD mice fed fish oil relative to the others for the measurement of total layers, in general and for the inner segment/outer segment layer, the outer nuclear layer, the outer plexiform layer, the inner nuclear layer, and the inner plexiform layer, in particular. The ganglion cell layer (GCL) density was increased in 5xFAD mice fed the DHASCO oil diet relative to the control (*p* < 0.05), suggesting a benefit of DHA supplementation on the number of viable ganglion cells. No positive staining was observed for β-amyloid plaques deposition or the neuroinflammatory marker, IBA1, corroborating previous findings in human AD retinas. Conversely, the internal retinal layers showed intense TAU immunostaining. Immnunostained TAU area was significantly reduced in 5xFAD mice fed a fish oil diet compared to control (*p* < 0.05), although the number of TAU-positive cells did not differ across diets (*p* > 0.05). The retinal protected integrity derived from the benefits of DHA supplementation found, either from fish oil or DHASCO oil, underscores the potential of retinal biomarkers as non-invasive indicators of cognitive decline and overall brain health, opening new avenues for investigating AD pathophysiology in the retina.

## 1. Introduction

Retinal health has emerged as a significant area of interest in Alzheimer’s disease (AD) research [1]. The retina, an extension of the central nervous system, reflects the pathological changes occurring in the brain. Retinal degeneration, characterized by the thinning of retinal layers, is increasingly recognized as an early indicator of neurodegenerative diseases, like AD. The easy accessibility of the retina and its direct connection to the brain makes it a promising non-invasive biomarker for detecting and monitoring AD progression. Changes in retinal thickness and cellular density, particularly in the neural layers, could provide valuable insights into cognitive decline and overall brain health [2]. The retina is a layered structure in the eye with ten distinct layers, most of them composed of neurons interconnected by synapses [3,4], that plays a crucial role in vision by converting light into neural signals. The cells subdivide into three basic cell types: photoreceptor cells, neuronal cells, and glial cells. The retina consists of ten distinct layers, arranged from the outermost (closest to the choroid) to the innermost (closest to the vitreous body): (1) the retinal pigment epithelium; (2) the inner segment/outer segment layer, also known as a layer of rods and cones; (3) the external limiting membrane; (4) the outer nuclear layer; (5) the outer plexiform layer; (6) the inner nuclear layer; (7) the inner plexiform layer; (8) the ganglion cell layer; (9) the nerve fibre layer; and (10) the inner limiting membrane [4].

The potential involvement of n-3 polyunsaturated fatty acids (n-3 PUFAs) in common retinal degeneration diseases, such as diabetic retinopathy, retinitis pigmentosa, and age-related macular degeneration, were evaluated before [5]. These essential nutrients, including alpha-linolenic acid (ALA, 18:3n − 3), eicosapentaenoic acid (EPA, 20:5n − 3), and docosahexaenoic acid (DHA, 22:6n − 3), are vital for cell membrane structure and function, playing a significant role in human health by supporting cardiovascular health, immune function, and neurological development. While clinical trials for established AD have not yet shown consistent benefits, the growing evidence suggests that the adequate intake of DHA is important for preventing cognitive impairment, especially in early AD stages, and in patients at a higher genetic risk for AD [6,7,8]. Yet, the essential role of DHA for protected retinal integrity in AD is far from being completely understood.

The 5xFAD mouse model is a traditional animal model for AD research [9,10]. It was designed to include multiple familial AD (FAD) mutations in order to increase β-amyloid accumulation, and to accelerate disease onset, reflecting the aggressive pathology observed in human FAD [9,10]. The 5xFAD transgenic mouse overexpresses at the same time the human amyloid precursor protein (APP) gene encoding three FAD mutations [the Swedish (KM670/671NL), the London (V717I) and the Florida (I716V), and the human presenilin1 (PSEN1) gene encoding two FAD mutations (the M146L and the L286V) [11]. These mutations potentiate the production and aggregation of β-amyloid peptides, contributing to the formation of amyloid plaques, and succeeding neurodegeneration [11], synaptic dysfunction, and cognitive impairment [10,12], mirroring the neuropathological features observed in human AD cases. The 5xFAD experimental model has great popularity and widespread use due to the fact that it is commercially available and cost-affordable, which diminish the price and time-period to obtain this strain. Additionally, AD hallmarks arise fast and strongly, reducing the expenses and time frame associated with animal housing, highly beneficial for the development of novel therapeutic and preventive AD strategies [13,14].

The 5xFAD transgenic mice were used to investigate the effects of DHA-enriched diets on retinal integrity and health. The study employed the measurement of retinal neural layer thickness, and the histological assessment of retinal injury, by means of immunohistochemistry staining for β-amyloid plaques aggregation, TAU protein count, and IBA1, as feature signals of AD progression. IBA1 is a protein widely used as a marker for microglia, the immune cells of the brain, and its expression is altered in AD. To complement this approach, we also analyzed the ganglion cell layer (GCL) for cell density. In AD, the GCL of the retina undergoes degeneration and thinning, marked by a significant loss of retinal ganglion cells (RGCs) reflecting similar pathological processes occurring in the brain. The loss of ganglion cells and their axons is a well-documented process in both human AD patients and animal models [15,16]. The retinal pathology is linked to the brain pathology and is considered a potential site for the non-invasive diagnosis and monitoring of AD [17,18,19].

## 2. Materials and Methods

### 2.1. Ethics Statement

This study followed European Union (Directive 2010/63/EU) guidelines and it was approved by the Ethics Review Board of CIISA/FMV, the Animal Welfare Committee of the National Veterinary Authority (Direção Geral de Alimentação e Veterinária (DGAV, Portugal, ref.: 20163/23-S accepted on 2 November 2023), and ORBEA/CIISA (ref.: 001/2023 accepted on 19 May 2023). The ARRIVE guidelines 2.0 have been strictly followed in this experiment (https://arriveguidelines.org/arrive-guidelines).

### 2.2. Mice, Experimental Design, and Diets

Forty five-week-old 5xFAD male mice were acquired from Charles River Laboratories (L’Arbresle, France). The mouse strain, B6SJL-Tg(APPSwFlLon,PSEN1M146LL286V)6799Vas/Mmjax, RRID, was provided by the Mutant Mouse Resource and Research Center (MMRRC) at The Jackson Laboratory, an NIH-funded strain repository. This strain was donated to the MMRRC by Robert Vassar, Ph.D., Northwestern University.

After arrival, mice were single housed at the Faculty of Veterinary Medicine animal facilities at room temperature of 22 ± 2 °C, relative humidity of 55 ± 5%, and a 12/12 h light–dark cycle (lights on at 8:00 a.m.) [20]. Mice were fed a standard maintenance purified diet (AIN-93M, Envigo, Barcelona, Spain) for the first two weeks to minimize stress. After this acclimation period, mice were allocated to five body weight-matched dietary groups with 8 animals each and fed isocaloric diets based on AIN-93M standard chow for rodents for 6 months. Each diet, with the exception of the control feed (without supplementation, the control group), contained a modified lipid fraction supplemented with 2% of the following: (1) linseed oil (LSO, rich in alpha-linolenic acid, the precursor of n3 LCPUFA pathway, the negative control); (2) cod liver oil (fish oil, FO rich in both DHA and EPA, the positive control); (3) *Schizochytrium* sp. microalga oil (Schizo) with 40% of DHA; (4) commercial DHASCO (DHASCO) with 70% of DHA. Oils were acquired from the following: linseed (flax) oil, ref.: 0140000, De Wit Speciality Oils, De Waal, The Netherlands; cod liver oil, ref.: CNP 7749945, Labsolve, José Manuel Gomes dos Santos, Lisbon, Portugal; NEWmegaTM Algae Oil, ref.: 0603640, De Wit Speciality Oils, The Netherlands; DHASCO, ref.: 6217-54-5, Dideu Industries, Xi’an, China. Diets were manufactured by Sparos company (Olhão, Portugal). The gross energy (kcal/kg) for each diet was 4001 ± 1.32 for control, 4067 ± 24.6 for LSO, 4041 ± 9.47 for FO, 4076 ± 2.65 for Schizo, and 4066 ± 4.00 for DHASCO. Ingredients in each diet were as follows (g/100 g DM, dry matter): casein (14.0), corn starch (46.6), maltodextrin (15.5), sucrose (10.0), cellulose (5.0), soybean oil (4.0), L-cystine (0.18), AIN-93M mineral mix (3.5), AIN-93M vitamin mix (1.0), choline bitartrate (0.25), and tert-butylhydroquinone (0.0008).

Ad libitum daily water and food intake were provided to mice throughout the experiment. Mice body weight and food intake were registered once a week. After 6 months of experimental trial, mice were fasted for 12 h to avoid the effect of either food in the gastrointestinal tract or the effect of newly absorbed nutrients. Then, mice were placed in a chamber, anesthetized with a mixture of 20% isoflurane in propylene glycol (*v*/*v*) for 30 sec, followed by decapitation applying a small rodents’ guillotine. The right eye was harvested and fixed by immersion in 10% neutral buffered formalin (Merck, VWR International, Radnor, PA, USA) for 24 h, and processed for paraffin embedding (Microscopy Histosec, Merck).

### 2.3. Fatty Acid Composition of the Experimental Diets

The fatty acid composition of DHA-enriched diets is shown in Table 1. Total lipids were extracted using Folch et al. [21] method. Fatty acid methyl esters (FAMEs) were obtained following Bandarra et al. [22] protocol by adding 5 mL of 5% acetyl chloride-methanolic solution. Tubes were placed in a water bath at 80 ◦C for 1 h. After tubes were cooled down, 1 mL of Milli-Q water and 2 mL of *n*-heptane were added to the tubes; next, tubes were agitated using the vortex, and the layers were separated by centrifugation at 3000× *g* for 3 min. The organic phase was isolated and filtered through anhydrous sodium sulphate. The final extract was analyzed by gas chromatography (GC, Scion 456-GC gas chromatograph, West Lothian, UK) prepared with a 30 m × 0.25 mm i.d, film thickness 0.25 μm, DB-WAX capillary column (Agilent Technologies, Santa Clara, CA, USA) with helium as the carrier gas, starting at 180 ◦C for 5 min, increasing to 220 °C at 4 °C/min, and holding for 24 min. The identification of fatty acids was based on the retention times of each fatty acid using PUFA-3 standard mix (Menhaden oil, Sigma-Aldrich, St. Louis, MO, USA) as internal reference.

### 2.4. Histology of the Retina

Serial tissue sections, 3 μm thick, were cut from each paraffin-embedded specimen using a Minot microtome (Leica RM2135, Nussloch, Germany). The sections were stained with classical Harris haematoxylin (Bio-Optica, Milan, Italy) and eosin-floxin (Bio-Optica) to assess morphology under a light microscope (Olympus BX51 equipped with Olympus DP21 microscope digital camera system, Olympus, Tokyo, Japan) to take photos. Of the ten retinal layers, the thickness of seven was assessed, individually or in combination, in triplicate per section at a magnification of ×400 using ImageJ software, version v1.54k/p/q, tools (https://fiji.sc/).

### 2.5. Quantification of Cell Density in the GCL of the Retina

Transverse sections of mice retina stained with classical Harris haematoxylin (Bio-Optica, Milan, Italy) and eosin-floxin (Bio-Optica) were visualized under a light microscope (Olympus BX51, Olympus) at 20× magnification. For each mouse, 6 different fields of view were captured, which were used as raw data for the cell counting pipeline. Each image was opened in the ImageJ and the scale was set according to the microscope magnification. Then, contrast was enhanced by 0.35% for each image. The image was then converted to 8-bit for easier processing and a colour threshold of 30–170 was set to isolate cells from the background. From the Binary tab in the Process bar, the Watershed function was performed to allow FIJI (version v1.54k/p/q) to identify cells within clusters. Using freeform selection, the RoI around the GCL layers was formed and the cells were calculated using the Analyze Particles function, while the area in µm^2^ was calculated by the Measure function. Freeform selection has a potential for human error but it was mitigated by taking six samples from every slide and then averaging them out to minimize the error and make any possible deviations uniform.

### 2.6. Retinal Immunohistochemistry

For immunohistochemistry assays, the retina sections were exposed to antigen retrieval with citrate buffer (pH 6.0) at 96 °C for 20 min with PTLink equipment (DAKO, LusoPalex, Carnaxide, Portugal). The sections were washed twice with distilled water for 5 min, treated with H_2_O_2_ to quench endogenous peroxidase activity (Envision FLEX Kit, catalogue #K8000, DAKO, LusoPalex, Carnaxide, Portugal) for 15 min, and then washed twice in PBS for 5 min. The sections were incubated with β-amyloid recombinant rabbit monoclonal antibody (H31L21) (1:500, Invitrogen, ThermoScientific, Waltham, MA, USA, catalogue #700254, RRID: AB_2532306), TAU recombinant rabbit monoclonal antibody (1:100, Invitrogen, ThermoScientific, catalogue #MA5-41098, RRID: AB_2898852), and IBA1 polyclonal antibody (1:100, Invitrogen, ThermoScientific, catalogue #PA5-27436, RRID: AB_2544912) for 1 h. After incubation, the sections were washed again twice in PBS for 5 min, incubated with donkey biotinylated anti-rabbit IgG (H+L) cross-absorbed secondary antibody (1:500, Invitrogen, ThermoScientific, catalogue #31458, RRID: AB_228213) at room temperature for 30 min, washed twice in PBS for 5 min, and developed with DAB (1:20, Envision FLEX Kit, DAKO, catalogue #K8000, LusoPalex, Carnaxide, Portugal) for 5 min. Then, sections were washed twice with distilled water for 5 min, stained with Harris haematoxylin (Bio-Optica) for 1 min, washed twice with distilled water for 5 min, dehydrated with increasing concentrations of ethanol, cleared with xylene, and covered with Entellan mounting reagent (Merck, VWR International, Radnor, PA, USA).

Immunohistochemistry with antibodies against Aβ, TAU, and IBA1 was performed to quantify β-amyloid plaques’ number and area, TAU-positive cells, and IBA1-positive cell number and area in 20 fields per section at ×400 magnification, using ImageJ software tools. The pictures were quantified in pixels following the histological protocol adapted from Christensen and Pike [23]. Antibody validation on positive control tissue (brain tissue) was performed at the time of the initial experiments, and the antibodies produced the expected staining pattern in these controls [24].

### 2.7. Statistical Analysis

Data analysis was carried out with Statistical Analysis System (SAS) software, version 9.1 [25], applying the generalized linear mixed (GLM) model. Data normality was verified by the Kolmogorov–Smirnov test, and the variance homogeneity was assessed by the Levene’s test. Significant multiple comparisons were carried out using the PDIFF option adjusted with the Tukey–Kramer’s test. The principal component (PC) analysis was performed, using the proc PRINCOMP of SAS, to assess relationships between variables. After data normalization, the analysis was based on the correlation matrix, and PCs were considered significant if they contributed more than 5% of the total variance. Data are presented as mean and SEM (standard error of the mean). A *p*-value less than 0.05 was considered to be statistically significant.

## 3. Results

### 3.1. Retinal Layer Thickness in the Retina of 5xFAD Mice Fed DHA-Enriched Diets

**Figure 1:**
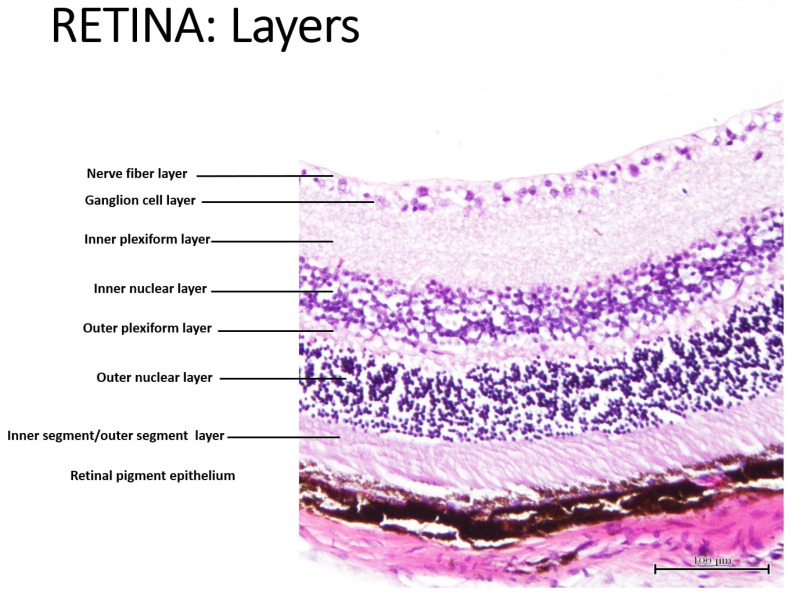
Illustrative representation of the major layers in the retina of 5xFAD mice (magnification of ×100, scale bar = 100 µm).

Figure 1 shows an illustration of the major retinal layers in 5xFAD mice.

**Figure 2:**
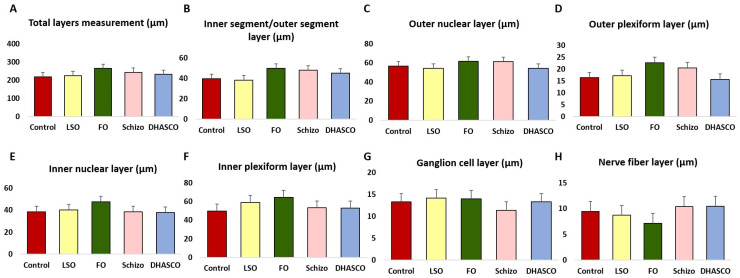
Measurement of retinal layers thickness (µm) in 5xFAD mice fed DHA-enriched diets. (**A**) total layers; (**B**) inner segment/outer segment layer; (**C**) outer nuclear layer; (**D**) outer plexiform layer; (**E**) inner nuclear layer; (**F**) inner plexiform layer; (**G**) ganglion cell layer; (**H**) nerve fibre layer. No statistically significant differences were found, *p* > 0.05.

Figure 2 illustrates the thickness measurements across the seven major retinal layers in 5xFAD mice fed DHA-enriched diets, individually or combined. While differences in the retinal layer thickness among the dietary groups did not reach statistical significance (*p* > 0.05), a consistent trend towards increased thickness was observed in fish oil-fed mice across multiple layers, including the total retina, the inner segment/outer segment layer, the outer nuclear layer, the outer plexiform layer, the inner nuclear layer, and the inner plexiform layer.

### 3.2. Ganglion Cell Layer (GCL) Density in the Retina of 5xFAD Mice Fed DHA-Enriched Diets

**Figure 3:**
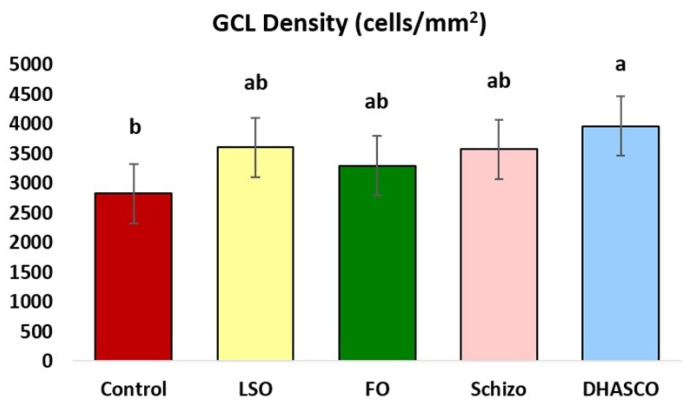
Ganglion cell layer (GCL) density in the retina of 5xFAD mice fed DHA-enriched diets. Different letters indicate statistical significances at *p* < 0.05.

Figure 3 shows the results for GCL density in the retina of 5xFAD mice under dietary interventions. The quantification of the retinal cell density (expressed in cells/mm^2^) can be used to assess Alzheimer’s disease prognosis and response to specific treatments. Herein, the GCL density was increased in 5xFAD mice fed the DHASCO oil diet relative to the control (*p* = 0.006). No other differences were observed for the remaining diets (*p* > 0.05).

### 3.3. Immunohistochemical Staining for β-Amyloid Plaques in the Retina of 5xFAD Mice Fed DHA-Enriched Diets

**Figure 4:**
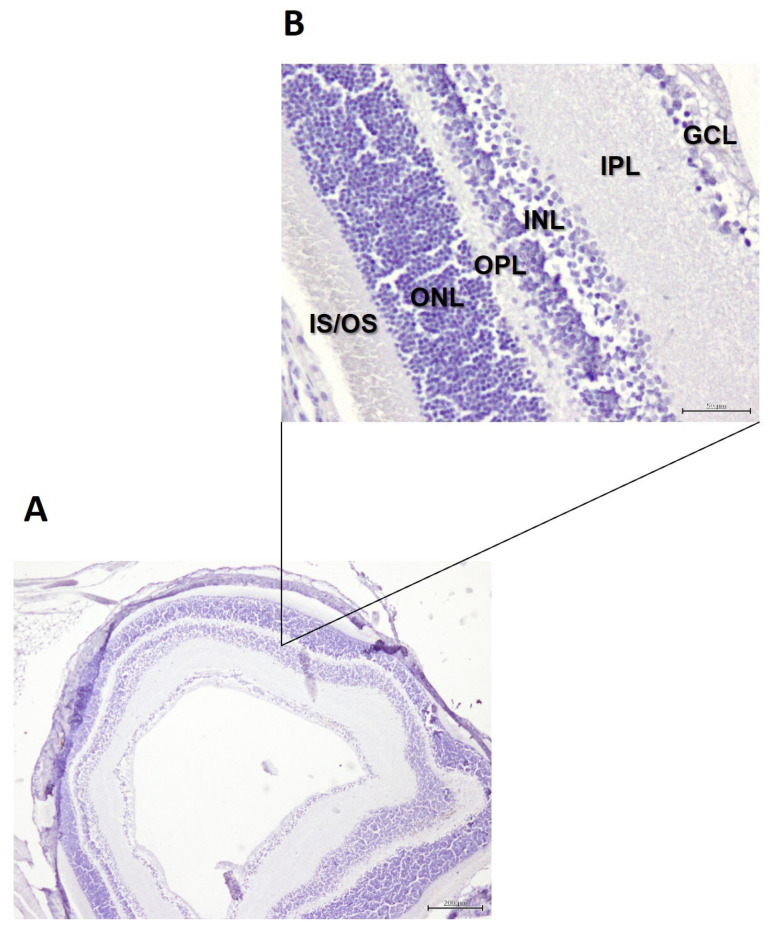
Illustrative images of immunohistochemical staining against β-amyloid plaques in the retina of 5xFAD mice fed DHA-enriched diets: (**A**) magnification of ×40, scale bar = 200 µm; (**B**) magnification of 200×, scale bar = 50 µm. IS/OS: Inner segment/outer segment layer; ONL: Outer nuclear layer; OPL: Outer plexiform layer; INL: Inner nuclear layer; IPL: Inner plexiform layer; GCL: Ganglion cell layer.

Immunohistochemistry using antibodies directed against β-amyloid plaques revealed no staining across all retinal layers (Figure 4A,B) in both control mice and mice fed DHA-enriched diets.

### 3.4. Immunohistochemical Staining for TAU in the Retina of 5xFAD Mice Fed DHA-Enriched Diets

**Figure 5:**
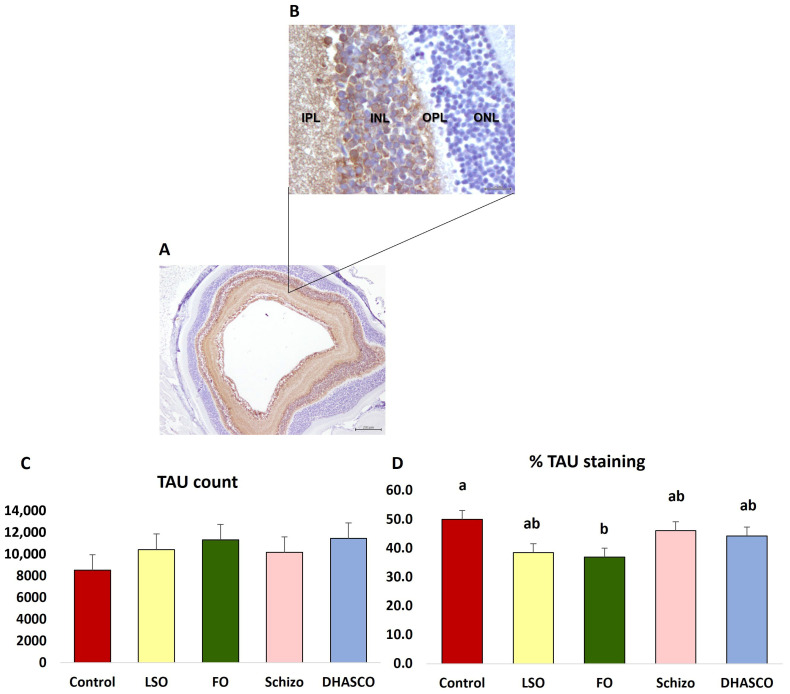
Representative immunohistochemical images of TAU staining in the retina of 5xFAD mice fed DHA-enriched diets: (**A**) magnification of ×40, scale bar = 200 µm; (**B**) magnification of 400×, scale bar = 20 µm. Graphical representations for the following: (**C**) TAU count; (**D**) % of TAU staining. ONL: Outer nuclear layer; OPL: Outer plexiform layer; INL: Inner nuclear layer; IPL: Inner plexiform layer. Different letters indicate statistical significances at *p* < 0.05.

Below are representative images of retinal cross-sections from 5xFAD mice following immunostaining with TAU antibody (Figure 5A,B) at two different microscope magnifications. Intense immunohistochemical staining for TAU was detected in the inner retinal layers, specifically within the inner nuclear layer, the inner plexiform layer, the ganglion cell layer, and the nerve fibre layer. In contrast, the outer retinal layers, including the photoreceptor layer (layer of rods and cones), the outer nuclear layer, and the outer plexiform layer showed no detectable TAU immunoreactivity (Figure 5A,B).

Moreover, immunostaining with TAU antibody showed no significant differences in the number of TAU-positive cells across the dietary groups (*p* = 0.571) (Figure 5C). However, the % of TAU immunostained area was significantly reduced in 5xFAD mice fed fish oil relative to the control group (*p* = 0.017) (Figure 5D).

### 3.5. Immunohistochemical Staining for IBA1 in the Retina of 5xFAD Mice Fed DHA-Enriched Diets

**Figure 6:**
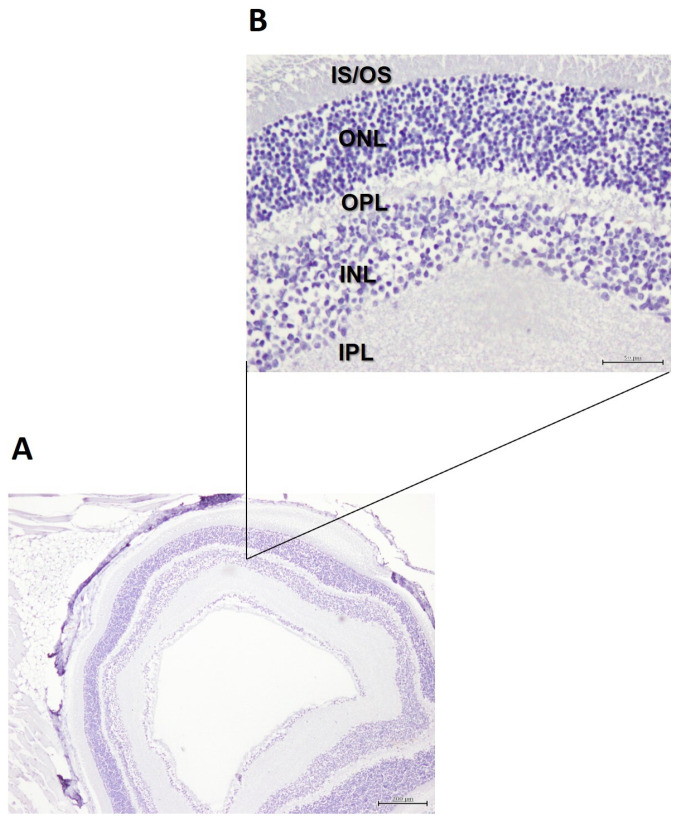
Illustrative images of immunohistochemical staining against IBA1 in the retina of 5xFAD mice fed DHA-enriched diets: (**A**) magnification of ×40, scale bar = 200 µm; (**B**) magnification of 200×, scale bar = 50 µm. IS/OS: Inner segment/outer segment layer; ONL: Outer nuclear layer; OPL: Outer plexiform layer; INL: Inner nuclear layer; IPL: Inner plexiform layer; GCL: Ganglion cell layer.

IBA1, a marker of microglial activation and thus an indicator of neuroinflammation, showed no detectable staining across any retinal layers in both control mice and mice fed DHA-enriched diets (Figure 6A,B).

### 3.6. Principal Component Analysis (PCA) Using Retinal Layer Thickness, GCL Density, and TAU Immunohistochemistry in the Retina of 5xFAD Mice Fed DHA-Enriched Diets

**Figure 7:**
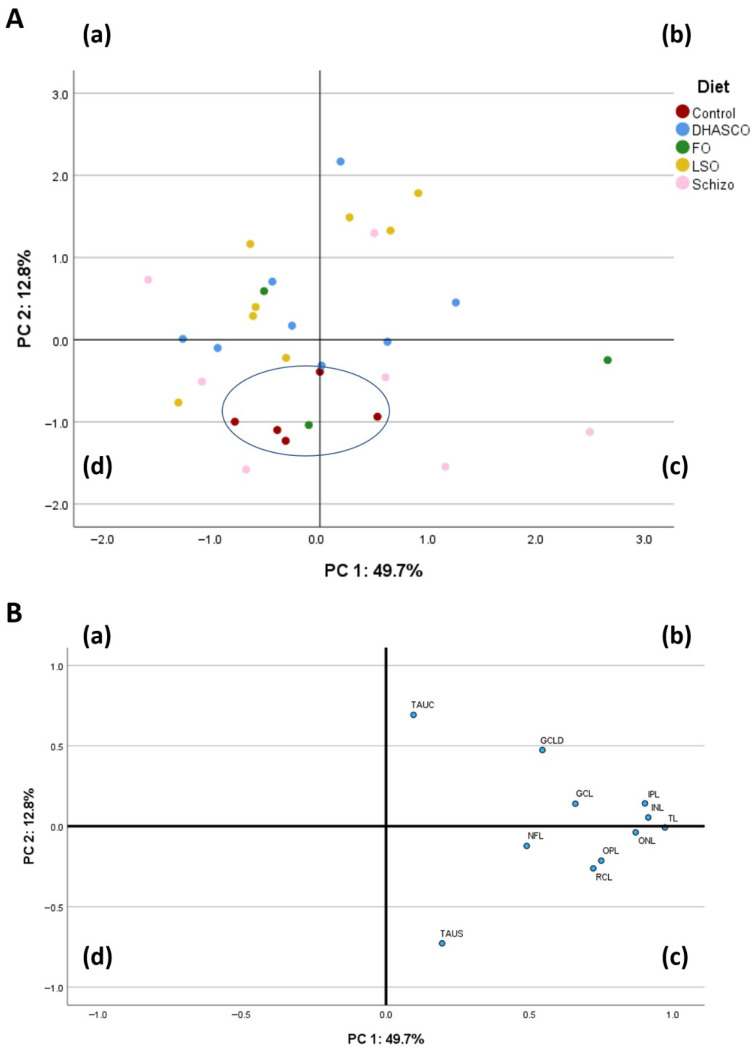
Loading plot of the first and second principal components (PCs) of the component score vectors (**A**) and pooled data (**B**) for the measurement of retinal layers thickness, TAU immunohistochemical staining, and ganglion cell layer (GCL) density in the retina of 5xFAD mice fed DHA-enriched diets: control diet; LSO, linseed oil diet; FO, fish oil diet; Schizo, *Schizochytrium* microalga oil diet; and DHASCO, commercial DHASCO oil diet. Blue circle in panel (**A**) indicates the discrimination of dietary groups.

To conclude our methodological analysis of retinal degeneration in 5xFAD mice fed DHA-enriched diets, we performed a principal component analysis (PCA) based on 11 variables in the correlation matrix (Figure 7). The PCA using the incorporated measurements of retinal layer thickness, TAU immunohistochemical staining, and GCL density was performed to describe the variability of the pooled data into two dimensions (Figure 7B). The score plot of the first two PC explained 62.5% of the total variability, with 49.7% for PC1 and 12.8% for PC2. The PC1 was characterized by variables with positive loadings, such as total layer (TL) thickness (0.973), inner nuclear layer (INL) thickness (0.915), inner plexiform layer (IPL) thickness (0.903), outer nuclear layer (ONL) thickness (0.871), outer plexiform layer (OPL) thickness (0.751), inner segment/outer segment layer thickness, also known as rods and cones layer (RCL) thickness (0.723), ganglion cell layer (GCL) thickness (0.660), ganglion cell layer density (GCLD) (0.545), and nerve fibre layer (NFL) thickness (0.491). Concerning the PC2, it was characterized by variables with positive loadings, such as TAU count (TAUC) (0.693), and by variables with negative loadings, such as percentage of TAU staining (TAUS) (−0.727). The score plot depicted in Figure 7A shows the location of the five experimental groups in the multivariate space of the first two PCs. The discrimination of dietary treatments was attainable with the control diet located in quadrants (c) and (d), and clearly separated from the other diets (Figure 7A). The LSO group was located in quadrants (a), (b), and (d) whereas the FO group was dispersed across quadrants (a), (c), and (d) (Figure 7A). The Schizo and DHASCO diets occupied all four quadrants, that is, (a), (b), (c), and (d) (Figure 7A).

## 4. Discussion

Recent evidence establishes a strong correlation between AD progression and irreversible neuronal lesions, with retinal degeneration emerging as a key aspect of this neurodegenerative condition [26,27,28]. The 5xFAD mouse model, which exhibits cognitive and spatial memory loss [9,10], displays amyloid pathology and neuroinflammation from an early age [29]. These mice also typically present memory deficits and learning impairment starting at around four months of age [11,24,30,31,32,33]. By the end of our experimental trial and at the time of euthanasia, 5xFAD transgenic mice were aged around eight months old.

In our study, mice fed a diet enriched with cod liver oil, that is, fish oil, showed a tendency, although not statistically significant, for increased total retinal thickness compared to the reference diet and the other experimental groups. A similar trend was observed for the following specific retinal layers: the inner segment/outer segment layer, the outer nuclear layer, the outer plexiform layer, the inner nuclear layer, and the inner plexiform layer. This finding suggests a protective putative effect against retinal degeneration, likely due to the potent antioxidant and anti-inflammatory properties inherent in fish oil [34,35]. Cod liver oil is known to contain appreciable amounts of vitamin A, also referred to as retinol [36,37], being considered a primary dietary source of this vitamin. The maintenance of retinal thickness, in particular in the neural layers, is pivotal for normal vision because these layers are more prone to degeneration in AD. This preservation is consistent with previous studies showing that dietary antioxidants effectively prevent retinal ganglion cell loss and help maintain retinal integrity in degenerative settings [38,39]. Furthermore, the slight improvement in retinal thickness in mice fed the fish oil diet highlights the potential of cod liver oil in maintaining retinal health. Because retinal changes associated with AD, such as neuronal loss, may occur before significant brain changes or symptoms appear, retinal biomarkers like retinal layer thickness ratios are being explored for early detection during the asymptomatic preclinical period of the disease. AD is associated with neuronal damage, leading to thinning of specific neural layers, like the retinal nerve fiber layer and ganglion cell layer. Comparing these neural layers to the total retinal thickness allows the detection of subtle signs of neurodegeneration that might be overlooked when assessing overall retinal thickness. A ratiometric analysis, expressed as the ratio of neural layers to total retinal thickness, has been proposed as a more sensitive indicator of retinal health in AD because it can help account for variations in overall retinal thickness that may be influenced by confounding factors such as age, gender, and ethnicity. Focusing on the ratio of neural layers allows researchers to isolate thinning caused by neurodegeneration in conditions like AD, providing a more specific marker than total thickness, which may also be affected by other systemic or anatomical factors. The ability to maintain or even increase this ratio through dietary strategies represents a significant step in defining non-invasive biomarkers for detection and monitoring AD progression. The Optical Coherence Tomography (OCT) is a non-invasive imaging technique often used to measure and segment individual retinal layers [40,41].

Aβ accumulation and the associated AD pathology have been extensively described in the retina [2,15,42,43,44,45], particularly in the inner retina, mostly inside the ganglion cell layer, and retinal blood vessels. These deposits increase with AD progression and correlate with brain changes, suggesting their potential as early biomarkers for AD. Overall, the accumulating evidence confirms the presence of AD-specific Aß species in the retina, suggesting a shared vulnerability of the retina and brain to AD pathological processes [46]. In our study, β-amyloid plaques were not detected in any retinal cell layer, in contrast to the pronounced Aβ deposition recently reported in the cerebral cortex of 5xFAD mice by de Mello-Sampayo et al. [24] using the same experimental settings. These contradictory findings may be explained by the stage of AD, particularly its milder manifestation during the early stages. In line with this, others reported immune alterations and morphological abnormalities in the brain and retina of AD mouse models prior to plaques deposition [32,47]. Additionally, a few studies in human patients have reported no Aβ accumulation in the retina [48,49], limiting the feasibility of detecting retinal biomarkers for early diagnosis.

The accumulation of microtubule-associated protein TAU isoforms (e.g., pTAU, neurofibrillary tangles-NFTs), collectively known as tauopathy, represents another hallmark of AD in the brain and is closely linked with neuronal injury and cognitive decline. There is growing evidence that TAU pathology occurs also in the retina of AD patients, as well as in murine models of AD, supporting the concept of retinal tauopathy, although the extent and nature of this pathology are still under investigation [50,51,52,53,54,55,56,57]. In fact, several retinal pTAU isoforms have been detected in the retina of AD patients. Not all studies consistently detect retinal pTAU and/or Aβ [48,49,50], and some studies still question whether retinal changes directly mirror cerebral TAU pathology. In this study, the internal retinal layers showed intense immunohistochemical staining for TAU protein. Although total TAU staining does not reflect pathological TAU species and our findings should therefore not be interpreted as evidence of tauopathy, we observed that the percentage of total TAU staining was significantly reduced in 5xFAD mice fed fish oil compared with those on the reference diet. Importantly, no differences were observed in the number of TAU-positive cells across diets, suggesting that fish oil affects TAU protein levels without altering the overall population of TAU-expressing cells.

More standardized protocols and larger well-controlled human studies are needed to confirm these findings. Moreover, 5xFAD mice do not normally develop TAU pathology including NFTs and neuritic plaques [33,58,59]. The 5xFAD have the potential to develop TAU pathology following injection with human TAU, or when TAU is overexpressed, and there is some evidence that TAU aggregates develop in the 5xFAD brain [60]. Retinal TAU alterations seen in 5xFAD mice, when viewed in the context of the recent literature, point to an unresolved question about the pathological relevance of total TAU levels in AD, and thus warrant further investigation.

Consistent with the observed distribution of retinal TAU levels in 5xFAD mice, mounting histological findings indicate subtle neurodegenerative changes, including some retinal atrophy and slight thinning across several cell layers. Numerous studies conducted in both humans and mouse models have demonstrated a complex pattern of AD-induced neurodegeneration spanning across all retinal layers, which may negatively impact a wide spectrum of retinal functions [42].

Retinal inflammation is a recognized component of AD, involving the activation of glial cells, and the accumulation of toxic proteins, like Aβ and TAU. Like stated before, the retina, being an extension of the central nervous system, can reflect pathological changes occurring in the brain [2,61], including inflammation [46]. In AD, microglia in both the brain and retina become hyperactivated [45,52] and release pro-inflammatory cytokines, such as TNF-α, IL-1β, and IL-6, which can exacerbate neuronal damage. In fact, there is evidence of elevated IBA1+ microgliosis and significant increases in reactive GFAP+ astrocytes [52,62], particularly notable at the retinal synapse-rich plexiform layers (IPL and OPL) in AD patients [62], suggesting a potential role of retinal microglia in excessive synaptic pruning. No evident signs of inflammation were observed in this study, as we were unable to quantify IBA1-positive cell count or staining area (%) due to the lack of successful antibody staining in the retinal sections. Despite prior validation of the antibody on positive control tissue, the absence of the IBA1 signal in these samples indicates that a technical failure likely occurred during this specific staining series. As a result, these data cannot be used to draw conclusions regarding microglial activation or neuroinflammation, and this represents a major experimental limitation of the study. Some signals would be expected, at least for the microglia population. In this respect, the authors should have considered alternative, validated antibodies which are widely used in similar studies. Including a non-AD control group would also help to determine whether the lack of IBA1 staining reflects a technical issue or disease-related changes. In fact. the extent of retinal microgliosis and macrogliosis closely correlates with increased retinal Aβ42 burden [62] suggesting that Aβ in the retina drives glial activation, as suggested in the AD brain. However, these microglia may exhibit impaired functionality in clearing amyloid deposits, contributing to their accumulation [62]. This explanation corroborates well our findings as amyloid deposition was not observed in the retina of 5xFAD mice.

The ganglion cell layer (GCL) in the mouse retina contains the cell bodies of retinal ganglion cells (RGCs), which are the output neurons of the visual system [63,64]. The ganglion cells are not uniformly distributed throughout the retina; while according to one study more than 8000 cells/mm^2^ were reported just temporal to the optic disc, as low as less than 2000 cells/mm^2^ were reported in the most dorsal retina [65]. For the whole retina, studies report an average of about 3000–3300 ganglion cells/mm^2^ in standard laboratory mice [66,67], in accordance with our numbers in 5xFAD mice. In this study, the GCL density was increased in mice fed the DHASCO oil diet (mean value of 3958 ganglion cells/mm^2^) relative to the reference diet (mean value of 2825 ganglion cells/mm^2^) but no other differences were observed. This finding points towards a significant health impact of the DHA-enriched diet from DHASCO oil in retinal protected integrity. In AD, the GCL of the retina, which contains RGCs, undergoes significant thinning and signs of neurodegeneration [68,69,70], reflecting similar pathological processes occurring in the brain. The degeneration of RGCs is characterized by a vacuolated, ‘frothy’ appearance of the cytoplasm, indicating neuronal damage, while the loss of ganglion cells and their axons is well-documented in both human AD patients and animal models [15,16]. Furthermore, this degeneration is correlated with cognitive decline and can be observed in both prodromal and established AD. Quantification of retinal cell density (expressed in cells/mm^2^) can be used to assess AD prognosis and monitor responses to specific treatments. We used FIJI, an open-source image processing platform that is essentially a distribution of ImageJ with batteries included, meaning it bundles the core ImageJ application with a large, curated collection of plugins specifically tailored for scientific image analysis [71]. Cell counting through FIJI has been validated through published studies [72]. Moreover, retinal imaging studies using OCT have shown that the retinal nerve fibre layer and ganglion cell layer thinning can be detected in AD patients, potentially serving as a biomarker for the disease [68,69,70]. These studies consistently show that both the ganglion cell layer and the retinal nerve fibre layer are thinner in individuals with AD compared to healthy controls. In fact, the degree of GCL thinning may be among the earliest detectable signs of AD [68,70], potentially preceding the onset of typical cognitive symptoms in AD. In essence, while the exact mechanisms are still being researched, it is believed that the degeneration of RGCs in AD is linked to the accumulation of amyloid β plaques and NFTs, similar to what is observed in the brain [68,69,70].

Our principal component analysis (PCA) clearly discriminated the reference mice from the other diets, clustering them in the bottom half of the loading plot. Considering all the parameters herein quantified, this outcome was expected, as most of the significant (or near-significant) differences observed were related to comparisons between the control group and other dietary groups (that is, the fish oil diet or DHASCO commercial oil diet).

## 5. Conclusions

AD has been increasingly linked to retinal changes as the retina is considered an extension of the central nervous system [2,61,73]. These retinal alterations correlate with cognitive decline and changes observed in the brain, suggesting that the retina could serve as a valuable site for detection and monitoring AD. Since retinal changes may precede cognitive decline and brain atrophy, retinal imaging could serve as an effective early biomarker for AD. Identifying biomarkers of AD will accelerate the understanding of its pathophysiology, facilitate screening and risk stratification, and support the development of new therapies. Herein, we exploit the measurement of retinal layer thickness as a source of potential biomarkers for AD. The slightly higher retinal thickness observed in mice fed a fish oil diet, although not statistically significant, both for the total retinal layers and specifically for the inner segment/outer segment layer, the outer nuclear layer, the outer plexiform layer, the inner nuclear layer, and the inner plexiform layer, supports its potential as a non-invasive biomarker for AD and warrants further investigation. In this regard, current imaging techniques, such as OCT, have revolutionized the investigation of the retinal changes in neurodegenerative disorders, namely AD, by providing highly detailed in vivo images of the retina [68,69,70], and thus should be applied in future studies.

Retinal degeneration in AD is commonly characterized by abnormal extracellular deposits of Aβ and intracellular TAU aggregates, leading to an intensified inflammatory response in the retina [26]. Although, we did not detect β-amyloid plaques accumulation or the microglial activation marker IBA1 in the retina by immunostaining, the percentage of TAU staining, as a proxy for TAU protein levels, was significantly reduced in mice fed fish oil compared to controls, corroborating our findings about the measurement of retinal layer thickness. In fact, DHA confers protection beyond generic antioxidant effects [74] on membrane fluidity, lipid raft composition, photoreceptor signalling, and synaptic plasticity. Unfortunately, the authors did not look for mechanistic explanations, due to limited sample sizes of the retina. In the future, it would be of interest to provide a mechanistic advancement regarding the observed protection of DHA, such as the DHA conversion to neuroprotection D1 (NPD1) [74], and downstream signalling (e.g., Bcl-2 family, ERK/Akt pathways) mediated by DHA itself or by its bioactive derivatives.

In summary, the neuroprotective effects of DHA supplementation in 5xFAD mice, via fish oil, which showed a consistent trend towards increased retinal layer thickness and reduced TAU aggregates, and DHASCO commercial oil, which increased GCL density, underscore the potential of these retinal biomarkers as non-invasive indicators of cognitive decline and brain health, and open new avenues for AD pathophysiology in the retina.

## Figures and Tables

**Table 1:** Fatty acid composition in experimental diets.

	Control	LSO	FO	Schizo	DHASCO
*Fatty acid composition (% of total fatty acids)*					
*Individual fatty acids*					
12:0	0.02 ± 0.04	0.01 ± 0.02	0.05 ± 0.00	0.16 ± 0.01	0.03 ± 0.03
13:0	0.18 ± 0.15	0.16 ± 0.02	0.20 ± 0.02	0.18 ± 0.03	0.18 ± 0.00
14:0	0.25 ± 0.02	0.18 ± 0.01	1.17 ± 0.03	2.04 ± 0.15	0.25 ± 0.02
16:0	12.39 ± 1.39	9.71 ± 0.01	11.39 ± 0.05	12.87 ± 0.18	9.62 ± 0.10
16:1n − 7	0.09 ± 0.08	0.10 ± 0.01	2.07 ± 0.08	1.88 ± 0.13	0.08 ± 0.07
17:0	0.07 ± 0.06	0.09 ± 0.00	0.10 ± 0.00	0.11 ± 0.00	0.05 ± 0.05
18:0	4.74 ± 0.22	4.70 ± 0.13	3.94 ± 0.04	3.84 ± 0.15	3.81 ± 0.02
18:1n − 9	22.72 ± 0.38	21.92 ± 0.41	21.20 ± 0.10	18.74 ± 0.43	18.47 ± 0.11
18:1n − 7	1.36 ± 0.04	1.15 ± 0.01	2.12 ± 0.03	2.63 ± 0.06	1.11 ± 0.01
18:2n − 6	50.67 ± 0.44	40.28 ± 0.10	38.12 ± 0.37	38.93 ± 0.59	40.51 ± 0.41
18:3n − 3	5.76 ± 0.18	20.07 ± 0.52	4.60 ± 0.05	4.46 ± 0.08	4.65 ± 0.06
18:4n − 3	nd	0.03 ± 0.05	0.54 ± 0.02	0.10 ± 0.03	nd
20:0	nd	0.30 ± 0.02	0.26 ± 0.01	0.30 ± 0.02	0.33 ± 0.00
20:1n − 9	0.17 ± 0.01	0.15 ± 0.01	2.92 ± 0.08	0.19 ± 0.02	0.15 ± 0.02
20:4n − 6	nd	nd	0.12 ± 0.01	0.04 ± 0.04	0.18 ± 0.00
20:5n − 3	nd	nd	2.59 ± 0.09	0.42 ± 0.02	0.12 ± 0.10
22:0	0.35 ± 0.02	0.29 ± 0.01	0.28 ± 0.00	0.30 ± 0.04	0.31 ± 0.01
22:5n − 6	nd	nd	nd	1.67 ± 0.05	3.37 ± 0.07
22:6n − 3	nd	nd	2.90 ± 0.09	10.01 ± 0.40	15.86 ± 0.37
*Sums and ratio of fatty acids*					
SFA	18.38 ± 1.44	15.44 ± 0.16	17.64 ± 0.03	19.98 ± 0.23	14.60 ± 0.14
MUFA	24.37 ± 0.43	23.35 ± 0.39	31.63 ± 0.16	23.52 ± 0.33	19.84 ± 0.11
PUFA	56.50 ± 0.51	60.43 ± 0.52	49.84 ± 0.20	55.75 ± 0.27	64.80 ± 0.15
n − 3 PUFA	5.76 ± 0.18	20.09 ± 0.54	11.24 ± 0.17	15.05 ± 0.43	20.71 ± 0.49
n − 6 PUFA	50.67 ± 0.44	40.30 ± 0.08	38.33 ± 0.36	40.65 ± 0.52	44.06 ± 0.35
n − 3/n − 6	0.11 ± 0.00	0.50 ± 0.01	0.29 ± 0.01	0.37 ± 0.01	0.47 ± 0.01

Control: AIN-93M diet; LSO: linseed oil diet; FO: fish oil diet; Schizo: *Schizochytrium* microalga oil diet; DHASCO: commercial DHASCO oil diet. nd: not detected. SFA: saturated fatty acid; MUFA: monounsaturated fatty acid; PUFA: polyunsaturated fatty acid. Results are expressed as the mean ± standard deviation (SD) of 3 replicates.

## Data Availability

Data generated within this study are included in the published article. All datasets produced during the experiment are available upon request from the corresponding author.
